# Effect of cholecalciferol on immune and vascular function in non-diabetic chronic kidney disease

**DOI:** 10.3389/fimmu.2025.1555304

**Published:** 2025-04-24

**Authors:** Kajal Kamboj, Aruna Pariki, Manphool Singhal, Anupam Lal, Sachin Naik, Vivek Kumar, Ashok Kumar Yadav, Vivekanand Jha

**Affiliations:** ^1^ Department of Nephrology, Postgraduate Institute of Medical Education and Research, Chandigarh, India; ^2^ Department of Radiodiagnosis and Imaging, Postgraduate Institute of Medical Education and Research, Chandigarh, India; ^3^ Department of Experimental Medicine and Biotechnology, Postgraduate Institute of Medical Education and Research, Chandigarh, India; ^4^ George Institute for Global Health, New Delhi, India; ^5^ School of Public Health, Imperial College, London, United Kingdom; ^6^ Prasanna School of Public Health, Manipal Academy of Higher Education, Manipal, India

**Keywords:** CKD, CVD, vitamin D deficiency, cholecalciferol, immune function, vascular function

## Abstract

**Background and aims:**

Vitamin D deficiency, widely prevalent in patients with chronic kidney disease (CKD) could play a role in the pathogenesis of cardiovascular disease (CVD) by causing alterations in endothelial and immune function. We investigated the change in immune and vascular functions following vitamin D supplementation in non-diabetic subjects with stage 3-4 CKD and vitamin D deficiency.

**Methods:**

In this single-arm study, non-diabetic CKD subjects aged 18–75 years, eGFR 15-60 ml/min/1.73m^2^, and serum 25-hydroxyvitamin D_3_ levels <20 ng/ml were enrolled. Enrolled subjects received a directly observed oral dose of 300,000 IU cholecalciferol at baseline and 8 weeks. Outcome assessments, including immunological, vascular, endothelial, inflammatory, and biochemical parameters, were measured at baseline and 16 weeks.

**Results:**

In total, 62 subjects were studied. The mean age was 44 ± 12 years with 58% men. TH1 cells decreased from 17% (9%, 27%) to 11% (6%, 16%) (p=0.002) and TH2 cells increased from 9% (5%, 16%) to 16% (10%, 27%) (p=0.001) after cholecalciferol treatment. A significant increase in mRNA expression of vitamin D-responsive genes (cathelicidin, IL-10, VDR, and CYP27B1) was observed. The levels of pro-inflammatory cytokines (IFN-γ, TNF-α, IL-23, and IL-6) decreased whereas anti-inflammatory cytokines (IL-4, IL-10, and IL-13) showed an increase. Cholecalciferol treatment improved flow-mediated dilatation (FMD): 8.2% (6.2%, 12.1%) at baseline to 14.1% (10.0%, 20.1%) at 16 weeks (p<0.001).

**Conclusions:**

This study confirms that cholecalciferol supplementation influenced immune function as it favored the TH2/TH1 phenotype, favorably affected the levels of inflammatory markers and mRNA expression of vitamin D responsive genes, and improved vascular function in CKD.

**Clinical Trial Registration:**

https://www.ctri.nic.in, identifier CTRI/2019/10/021494.

## Introduction

Cardiovascular disease (CVD) is the predominant comorbidity of chronic kidney disease (CKD). A majority of patients with CKD experience cardiovascular complications prior to advancing to kidney failure (1). Vitamin D deficiency is prevalent amongst those with CKD and has been linked with mortality in both early and advanced CKD, including in those on dialysis (2–4).

There are several experimental reports that indicate that vitamin D affects innate and adaptive immunity (5, 6); however, the regulatory direction is opposite to each other, i.e., vitamin D inhibits adaptive immunity while promoting innate immunity (7). The inhibition of immune responses with 1,25-dihydroxy vitamin D_3_ [1,25 (OH)_2_D_3_] is orchestrated by type 1 helper (TH1) cells, known for their capacity to generate inflammatory cytokines (8). This suppression leads to shifting towards type 2 helper (TH2) development by enhancing cytokines associated with TH2 cells. Vitamin D additionally triggers the production of regulatory T (Treg) cells, pivotal for inflammation suppression (9). 1,25 (OH)_2_D_3_ has also been reported to suppress the development of TH17 cells (10). Despite the potential significance of vitamin D across numerous immune functions, the precise mechanisms, including the connection between innate and adaptive immunity, remain unclear.

Further, it has been already demonstrated that cholecalciferol supplementation improves vascular and endothelial function in CKD, as assessed using flow-mediated dilatation (FMD) and pulse wave velocity (PWV) along with endothelial biomarkers (11–14), immune function (T helper cells), and inflammatory markers (anti- and pro-inflammatory cytokines) (15, 16). Immune dysregulation is also recognized as a contributor to endothelial damage, vascular dysfunction, and the development of atherosclerotic plaques, ultimately leading to CVD (17). No study to date has investigated the effects of cholecalciferol on the immunological axis in conjunction with cardiovascular outcomes.

We hypothesized that improvement in immune function after supplementation of cholecalciferol is linked with favorable changes in vascular function and tested this by evaluating the change in immune function and vascular function at 16 weeks after supplementation with cholecalciferol in non-diabetic subjects with stage 3–4 CKD and vitamin D deficiency.

## Methods

### Study setting

This was a single-center, prospective interventional study conducted at the Postgraduate Institute of Medical Education and Research (PGIMER), Chandigarh, India. The study protocol was approved by the Institute’s Ethics Committee (IEC) (No: NK/6731/PhD/930 dated 01/12/2020). The study was registered in the clinical trial registry of India (CTRI/2019/10/021494). Enrolment of study subjects started in March 2021 and was completed in December 2022.

### Study population

Subjects with pre-dialysis CKD attending the outpatient clinic of the Department of Nephrology at PGIMER, Chandigarh, India were screened for enrolment. Clinically stable patients between the ages of 18–75 years with an estimated glomerular filtration rate (eGFR) using the creatinine-based Chronic Kidney Disease Epidemiology collaboration equation (CKD-EPIcr_2009_) of 15-60 ml/min/1.73m^2^ and serum 25-hydroxyvitamin D_3_ levels <20 ng/ml were enrolled. Patients receiving immunosuppressive therapy; those with a diagnosis of diabetes mellitus, chronic liver disease, primary hyperparathyroidism, sarcoidosis, or malignancy; those with a history of having been treated for hypercalcemia due to any cause; those anticipated to need long-term kidney replacement therapy within 6 months; those with poor functional status or life expectancy <1 year as judged by the treating physician; pregnant patients in case of women; those with a smoking history, hemoglobin <8 g/dl, serum calcium >9.5 mg/dl, and a history of having received any type of organ transplantation; and those who had received vitamin D supplementation in previous 30 days were excluded. All patients provided written informed consent before enrolment. The study adhered to the Declaration of Helsinki.

### Intervention and follow-up

The demographic and clinical data were recorded at enrolment. Subjects received a directly observed oral dose of 300,000 IU cholecalciferol (Cipcal^®^D3, Cipla) at baseline and at 8 weeks. The final follow-up visit took place at 16 weeks. Blood and spot urine samples were collected at baseline and at 16 weeks and stored at -80°C for further analysis.

### Outcomes

The primary outcome was the change in circulating T cell subsets (TH1, TH2, TH17, and Treg) at 16 weeks after cholecalciferol supplementation. Secondary outcomes were changes in the expression of vitamin D responsive genes (cathelicidin, IL-10, VDR, and CYP27B1), changes in vascular function [FMD, nitro-glycerine mediated dilatation (NMD), and pulse weve velocity (PWV)], changes in the levels of endothelial function biomarkers [E-Selectin, intercellular adhesion molecule (ICAM) and vascular cell adhesion molecule (VCAM)], changes in the levels of pro-inflammatory (IFN-γ, TNF-α, IL-1β, IL-17, and IL-2) and anti-inflammatory cytokines (IL-4, IL-10, and IL-13), and association between the change in FMD and NMD and the change in circulating T cell subsets at 16 weeks.

### Measurements

Hemoglobin, calcium, phosphate, lipid profile, urine albumin, and creatinine were measured at the central laboratory of PGIMER, Chandigarh. Serum intact parathyroid hormone (iPTH) and 25-hydroxyvitamin D_3_ levels were analyzed using a Cobas^®^ 8000 modular analyzer (Roche Diagnostics, Mannheim, Germany). Serum 1,25 (OH)_2_ D_3_ was analyzed by sandwich enzyme-linked immunosorbent assay (ELISA) (IDS Ltd, Fountain Hills, AZ, USA; Cat No. AC-62F1) according to the manufacturer’s instructions.

### Immune function

Immune function was assessed through the immunophenotyping of T cell subsets (TH1, TH2, TH17, and Treg) and mRNA analysis of vitamin D-responsive genes (cathelicidin, IL-10, VDR, and CYP27B1) in peripheral blood mononuclear cells (PBMCs) at baseline and 16 weeks. The T cell subsets were analyzed using flow cytometry. T cell subset panels (**Supplementary Table 1**) were prepared with antibodies conjugated to different fluorescent markers (Becton Dickinson Biosciences, Franklin Lakes, New Jersey, USA). The acquisition of cells was performed on a BD-FACS Aria and the percentage of cells was analyzed using FACS-Diva software (Becton Dickinson, Franklin Lakes, New Jersey, USA).

The mRNA expression of vitamin D-responsive genes was analyzed by extracting total RNA using an RNeasy® Mini Kit (Qiagen, Hilden, Germany, Cat. No. 74104), from PBMCs. cDNA was prepared using a SuperScript™ IV First-Strand cDNA Synthesis Kit (Invitrogen, Thermo-fisher, Waltham, MA, USA, Cat No. 18091050), and quantitative real-time polymerase chain reaction (qRT-PCR) was carried out for cathelicidin, IL-10, VDR, and CYP27B1. A real-time PCR machine (ABI Biosystem Prism 7500, ABI Biosystem, Foster City, CA, USA) was used for analysis and a Taqman expression assay (ABI Biosystem, Foster City, CA, USA) (**Supplementary Table 2**) was used for mRNA expression analysis using the relative quantification method.

### Inflammatory markers

Serum levels of pro-inflammatory cytokines (IFN-γ, TNF-α, IL-1β, IL-17, and IL-2) and anti-inflammatory cytokines (IL-4, IL-10, and IL-13) were measured at baseline and 16 weeks using a Luminex Multiplex Array (Milliplex^®^, Merck, Darmstadt, Germany, Cat. No. HCYTA-60K) as per the manufacturer’s protocol. IL-23 and IL-6 were analyzed using ELISA kits (Quantikine solid-phase sandwich ELISA; R&D Systems^®^, Minneapolis, MN, USA; Cat No. D2300B and D6050B respectively). Human serum C-reactive protein (hsCRP) was analyzed using (Calbiotech Inc., CA, USA; Cat No. CR375C) as per the manufacturer’s protocol.

### Vascular function

FMD, NMD, and PWV were measured at baseline and 16 weeks. FMD and NMD were conducted in the morning in a fasting state at an ambient temperature of 20°–25°C using a Philips IU22 xMatrix ultrasound system (Philips, Cambridge, MA, USA) and Philips IU22 L12–5 linear transducer with 5–12 MHz as described previously (18). Carotid-femoral PWV was measured at the same time (18).

Endothelial function markers (E-Selectin, ICAM, and VCAM) were estimated from plasma samples using ELISA kits (Quantikine^®^ immunoassay, R&D systems, Minneapolis, USA; Cat No. DSLE00, DCD540, DVC00 respectively) as per the manufacturer’s protocol.

### Sample size and statistical analysis

Based on data regarding change in Tregs with vitamin D supplementation (19) (mean change 0.6%), a sample size of 65 patients was required to detect this degree of change with 80% power and an alpha of 0.05 assuming a 15% dropout rate.

All enrolled subjects with non-missing outcome data were included in the analysis. Descriptive statistics were used to describe the characteristics of study subjects. Data are expressed as mean ± SD or median (IQR) for continuous variables and number and percentage for categorical variables as appropriate. A paired t-test and Wilcoxon signed-rank test, as appropriate, were used to compare the effect of cholecalciferol supplementation on various parameters including vascular function, immune function, and inflammatory markers in patients with CKD. For adjustment of FMD with baseline vitamin D, age, sex, SBP and DBP, and CVD, a linear mixed effect model was used. Correlation analysis between the change in markers of immune function and markers of vascular function was conducted using Spearman’s rank correlation. A P-value <0.05 was considered significant. Statistical Package for the Social Sciences (SPSS Inc., Chicago, IL, USA, version 23.0) and GraphPad Prism 9.0 (San Diego, California, USA) were used for the analysis.

## Results

**Figure 1:**
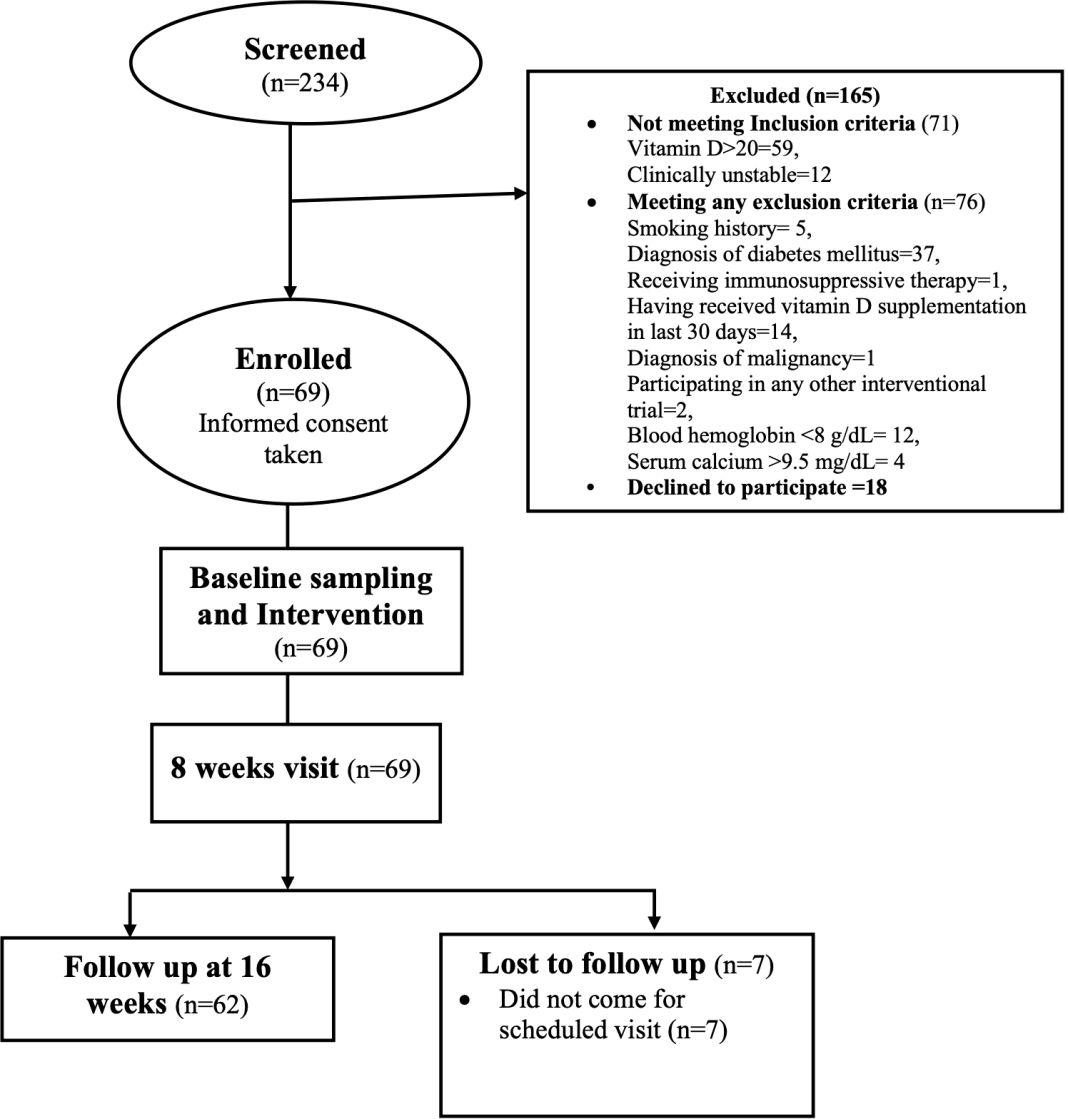
CONSORT diagram showing study flow at baseline and follow-up.

A total of 234 subjects were screened between March 2021 and December 2022: 165 were excluded—71 for not meeting inclusion criteria and 76 for meeting one of the exclusion criteria. A total of 87 subjects were invited to participate, of whom 18 declined. Finally, 69 subjects were enrolled after obtaining informed consent (**Figure 1**) and 62 subjects completed the 16-week follow-up. The mean age of the study subjects was 44 ± 12 years and 58% were men. The cause of CKD was dominated by unknown causes (21, 34%) followed by chronic interstitial nephritis (15, 24%), glomerulonephritis (8, 13%), congenital anomalies of the kidney and urinary tract (CAKUT) (3, 5%), polycystic kidney disease (PKD) (2, 3%), and others (13, 21%).

Serum creatinine significantly increased at the 16-week follow-up from 2.3 ± 0.8 mg/dl to 2.8 ± 2.0 mg/dl (p<0.001) and the eGFR decreased from 32 (23, 46) ml/min/1·73 m^2^ to 30 (18, 41) ml/min/1·73 m^2^ (p=0.002). Serum 25-hydroxyvitamin D_3_ levels showed a significant increase from 13 ng/ml (9 ng/ml, 16 ng/ml) at baseline to 38 ng/ml (27 ng/ml, 49 ng/ml) at follow-up (mean difference 25 ng/ml, 95% CI: 21 to 30 ng/ml, p<0.001). The levels of 1, 25 (OH)_2_ D_3_ also improved: 27 pg/ml (18 pg/ml, 47 pg/ml) at baseline vs 45 pg/ml (26 pg/ml, 79 pg/ml) at follow-up (mean difference: 34 pg/ml, 95 CI%: 14 to 53 pg/ml, p=0.001). There was a significant decrease in alkaline phosphatase (p=0.02) (**Table 1**).

**Table 1:** Comparison of the clinical, biochemical, and vascular function of study subjects at baseline and 16-week follow-up (N=62).

Characteristic	Baseline	Follow-up (16 weeks)	Mean difference (95% CI)	P-value
Hemoglobin (g/dl)	11.6 ± 1.9	11.6 ± 1.9	-0.04 (-0.42 to 0.33)	0.50
Serum creatinine (mg/dl)	2.3 ± 0.8	2.8 ± 2.0	0.47 (0.10 to 0.85)	<0.001
eGFR (ml/min/1·73 m^2^)	32 (23, 46)	30 (18, 41)	-2.50 (-5.00 to 0.01)	0.002
Body mass index (kg/m^2^)	24.4 ± 4.6	24.6 ± 4.7	0.18 (-0.17 to 0.52)	0.18
Serum urea (mg/dl)	55.8 (39.9, 71.9)	60.2 (48.3, 84.7)	10.8 (5.7 to 15.9)	<0.001
Total cholesterol (mg/dl)	155 (138, 200)	163 (138, 191)	-3.8 (-18.4 to 10.8)	0.98
Triglycerides (mg/dl)	161(126, 211)	15 (121, 195)	-15.1 (-57.9 to 27.6)	0.86
LDL-C (mg/dl)	94 (70, 122)	96 (74, 123)	-0.6 (-8.8 to 7.7)	0.82
HDL-C (mg/dl)	40 (35, 50)	41 (35, 52)	0.4 (-3.9 to 4.8)	0.42
Calcium (mg/dl)	8.9 (8.5, 9.2)	9.0 (8.7, 9.3)	0.06 (-0.10 to 0.22)	0.61
Inorganic phosphorous (mg/dl)	3.7 (3.2, 4.1)	3.7 (3.12, 4.3)	0.14 (-0.11 to 0.39)	0.23
Uric acid (mg/dl)	7.2 (5.9, 8.2)	7.2 (6.3, 8.8)	0.14 (-0.27 to 0.54)	0.49
Alkaline phosphatase (U/L)	96 (79,115)	91 (77, 113)	-4.7 (-11.1 to 1.8)	0.02
Serum iPTH (pg/ml)	159 (94, 280)	161 (91, 239)	-35.7 (-66.9 to -4.4)	0.06
Urine albumin creatinine ratio (mg/g)	7930 (1475, 15462)	5010 (745, 5010)	1787 (-4440 to 865)	0.18
Serum 25-hydroxyvitamin D_3_ (ng/ml)	13 (9, 16)	38 (27, 49)	25 (21 to 30)	<0.001
1,25 (OH)_2_D_3_ (pg/ml)	27 (18, 47)	45 (26, 79)	34 (14 to 53)	0.001
Flow-mediated dilatation (%)	8.2 (6.2, 12.1)	14.1 (10.0, 20.1)	6.0 (4.0 to 7.9)	<0.001
Nitroglycerin-mediated dilatation (%)	10.7 (6.1, 14.7)	13.8 (9.9, 18.9)	4.4 (2.1to 6.7)	<0.001
Pulse wave velocity (m/s)	8.2 (7.1, 10.2)	7.7 (7.1, 9.4)	-0.43 (-0.90 to 0.04)	0.07

Data presented as mean ± standard deviation or median (25^th^ and 75^th^ percentile) or mean difference (95% confidence interval) as appropriate. P-values <0.05 were considered significant.

1,25 (OH)_2_ D3, 1,25 dihydroxy vitamin D; eGFR, estimated glomerular filtration rate; HDL-C, high-density lipoprotein cholesterol; iPTH, intact parathyroid hormone; LDL-C, low-density lipoprotein cholesterol.

### Effect of cholecalciferol on immune function in patients with CKD

**Table 2** shows the changes in different T cell populations at baseline and at 16 weeks. TH1 cells (CD3^+^CD4^+^IFNγ^+^) decreased from 17% (9%, 27%) to 11% (6%, 16%) (p=0.002) and TH2 (CD3^+^CD4^+^IL4^+^) cells increased from 9% (5%, 16%) to 16% (10%, 27%) (p=0.001). However, TH17 (CD3^+^CD4^+^IL17A^+^) and Treg (CD3^+^CD4^+^CD25^+^CD127^low^FOXP3^+^) cell populations showed no significant change (p=0.17 and p=0.99).

**Table 2:** Effect of cholecalciferol supplementation on T cell phenotype at 16-week follow-up.

T cell subpopulation	T cell marker	Baseline (% cells)	Follow up (% cells)	P-value
TH1 cells	CD3^+^CD4^+^CXCR3^+^	29 (17, 43)	24 (10, 37)	0.13
	CD3^+^CD4^+^Tbet^+^	12 (7, 20)	13 (8, 19)	0.61
	CD3^+^CD4^+^IFNγ^+^	17 (9, 27)	11 (6, 16)	0.002
TH2 cells	CD3^+^CD4^+^IL4^+^	9 (5, 16)	16 (10, 27)	0.001
	CD3^+^CD4^+^STAT6^+^	8 (4, 14)	13 (9, 20)	0.001
	CD3^+^CD4^+^GATA3^+^	16 (10, 23)	18 (12, 30)	0.09
	CD3^+^CD4^+^CCR4^+^CCR6^-^	14 (10, 20)	13 (9, 16)	0.10
TH17 cells	CD3^+^CD4^+^IL17A^+^	5 (1, 11)	8 (4, 12)	0.17
	CD3^+^CD4^+^RORγt^+^	12 (4, 19)	9 (5, 15)	0.16
	CD3^+^CD4^+^CCR4^+^CCR6^+^	9 (4, 13)	7 (4, 12)	0.57
Treg cells	CD3^+^CD4^+^CD25^+^	15 (8, 24)	16 (9, 23)	0.38
	CD3^+^CD4^+^CD25^+^CD127^low^FOXP3^+^	2.7 (1.8, 3.7)	2.8 (1.9, 3.5)	0.99

Data presented as median (25^th^, 75^th^ percentile). TH1, T helper 1 cell population; TH2, T helper 2 cell population; TH17, T helper 17 cell population; Treg, T regulatory cell population.

**Figure 2:**
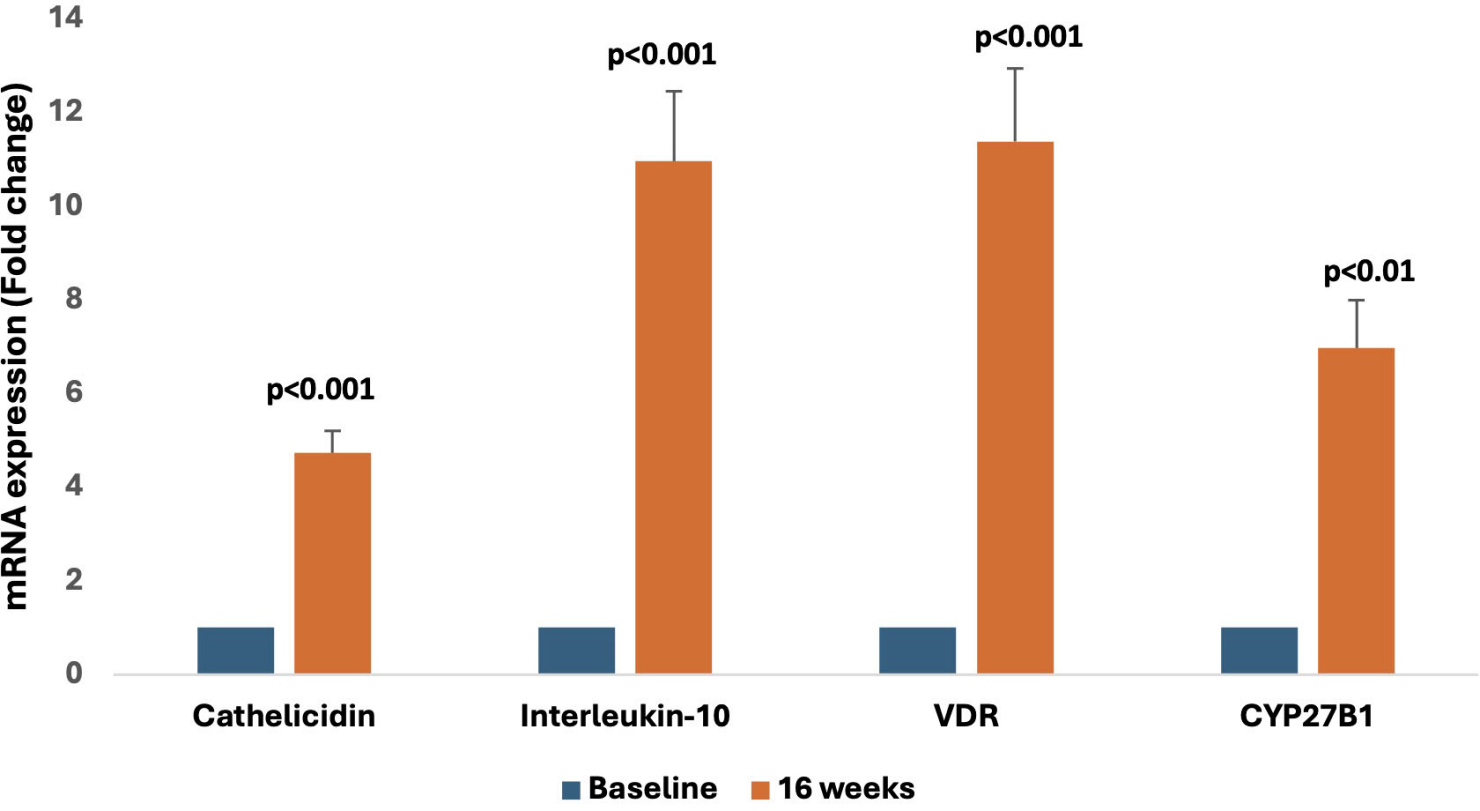
Representative graphs showing relative mRNA expression of vitamin D-responsive genes (cathelicidin, Interleukin-10, VDR, and CYP27B1) at the 16-week follow-up compared to baseline.

There was a significant change in the expression of vitamin D-responsive genes as shown by the increase in cathelicidin (4.7 fold, p<0.001), IL-10 (11 fold, p<0.001), VDR (11.4 fold, p<0.001), and CYP27B1(7 fold, p<0.01) levels respectively at 16 weeks as compared to baseline (**Figure 2**).

### Effect of cholecalciferol on inflammatory markers

**Figure 3:**
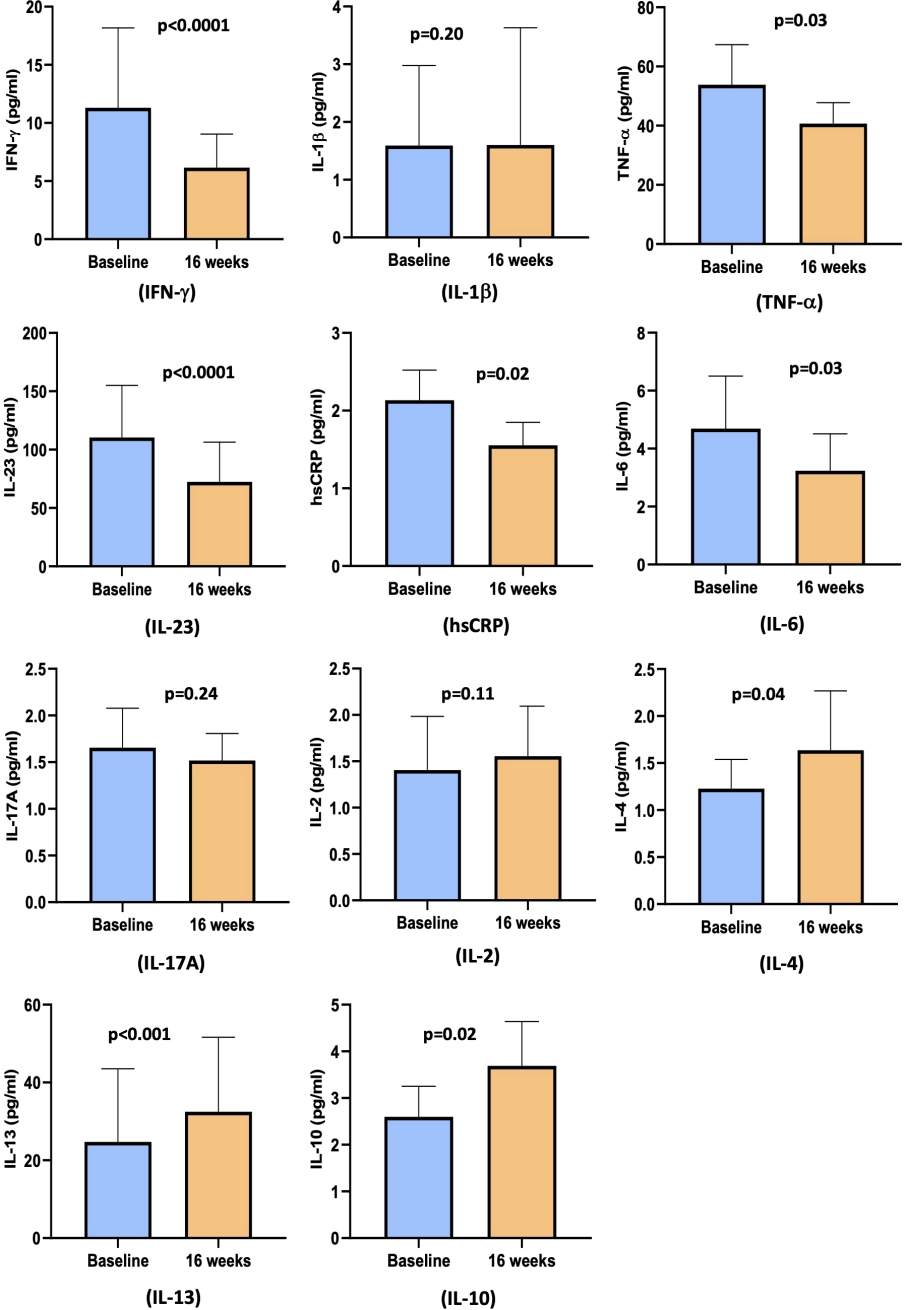
Bar diagram representing pro- and anti-inflammatory cytokines levels at baseline and at 16 weeks: IFN-γ, IL-1β TNF-α, IL-23, hsCRP, IL-6, IL-17A, IL-2, IL-4, IL-13, and IL-10.

As shown in **Figure 3**, cholecalciferol treatment significantly reduced the levels of the pro-inflammatory cytokine IFN-γ [4.9 pg/ml (1.9 pg/ml, 9.0 pg/ml) vs. 2.0 pg/ml (1.3 pg/ml, 6.1 pg/ml), p<0.0001], TNF-α [45 pg/ml (26 pg/ml, 61 pg/ml) vs. 38 pg/ml (24 pg/ml, 48 pg/ml), p=0.03], IL-23 [74 pg/ml (67 pg/ml, 94 pg/ml) vs. 38 pg/ml (13 pg/ml, 59 pg/ml), p<0.0001], hsCRP [1.5 pg/ml (0.9 pg/ml, 3.7 pg/ml) vs. 1.1 pg/ml (0.7 pg/ml, 2.2 pg/ml), p=0.02], and IL-6 levels [2.2 pg/ml (1.2 pg/ml, 4.4 pg/ml) vs. 1.8 pg/ml (0.7 pg/ml, 3.8 pg/ml) pg/ml, p=0.03]. However, no change was observed in the levels of IL-1β [1.6 pg/ml (0.1 pg/ml, 3.0 pg/ml) vs. 1.6 pg/ml (1.6 pg/ml, 3.6 pg/ml), p=0.20], IL-17A [1.7± 1.7 pg/ml vs. 1.5± 1.1 pg/ml, p=0.24], and IL-2 [0.8 pg/ml (0.5 pg/ml, 1.4 pg/ml) vs. 0.9 pg/ml (0.6 pg/ml, 1.5 pg/ml), p=0.11].

The circulating levels of anti-inflammatory markers showed a significant change with cholecalciferol supplementation at 16 weeks. There was an increase in the levels of IL-4 [0.8 pg/ml (0.5 pg/ml, 1.5 pg/ml) vs. 0.9 pg/ml (0.6 pg/ml, 1.5 pg/ml), p=0.04], IL-10 [2.0 pg/ml (0.8 pg/ml, 3.2 pg/ml) vs. 2.6 pg/ml (1.3 pg/ml, 4.4 pg/ml), p=0.02], and IL-13 [6.4 pg/ml (6.4 pg/ml, 14.5 pg/ml) vs. 8.4 pg/ml (6.4 pg/ml, 28.1 pg/ml), p=0.0004] (**Figure 3**).

### Effect of cholecalciferol on vascular function

**Figure 4:**
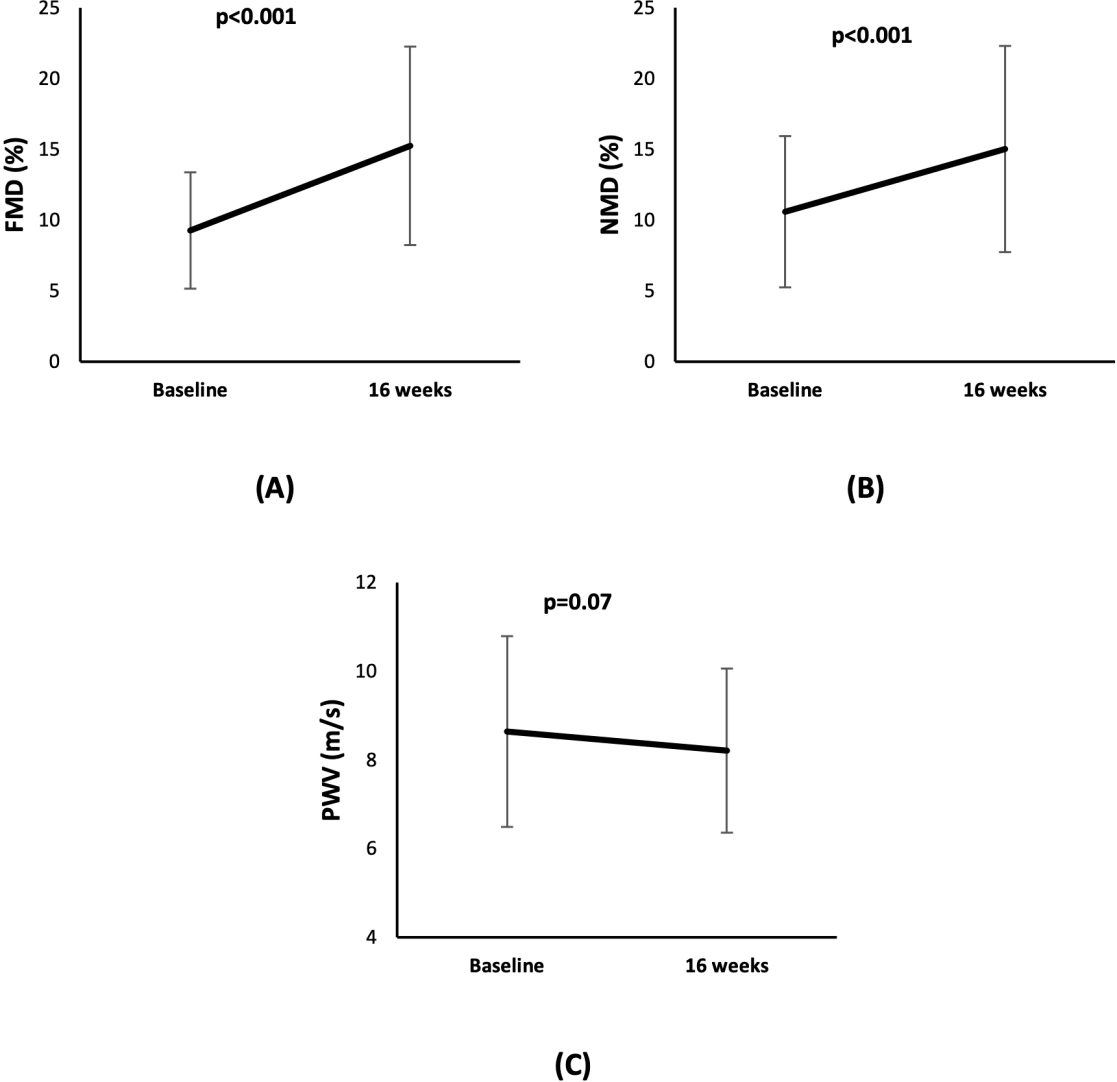
Line diagram showing the effect of cholecalciferol on levels of vascular function markers **(A)** FMD **(B)** NMD, and **(C)** PWV at baseline and at 16 weeks.

The FMD improved from 8.2% (6.2%, 12.1%) at baseline to 14.1% (10.0%, 20.1%) at 16 weeks (mean diff: 6.0%, 95% CI: 4.0% to 7.0%, p<0.001). Similarly, there was a significant change in NMD from 10.7% (6.1%, 14.7%) at baseline to 13.8% (9.9%, 18.9%) at follow-up (mean diff: 4.4%, 95% CI: 2.1% to 6.7%, p<0.001). PWV showed a trend towards improvement after cholecalciferol treatment from 8.2 m/s (7.1 m/s, 10.2 m/s) at baseline to 7.7 m/s (7.1 m/s, 9.4 m/s) at the follow-up at 16 weeks (mean diff: -0.43 m/s, 95%CI: -0.90 to 0.04 m/s, p=0.07), but the change was not significant (**Table 1**) (**Figure 4**). In the linear mixed model, after adjustment for baseline vitamin D, age, sex, SBP and DBP, and CVD, FMD showed significant improvement with an estimated mean difference of 3.3% and 95% CI of 0.5% to 6.1%. There was no significant change in NMD and PWV.

**Figure 5:**
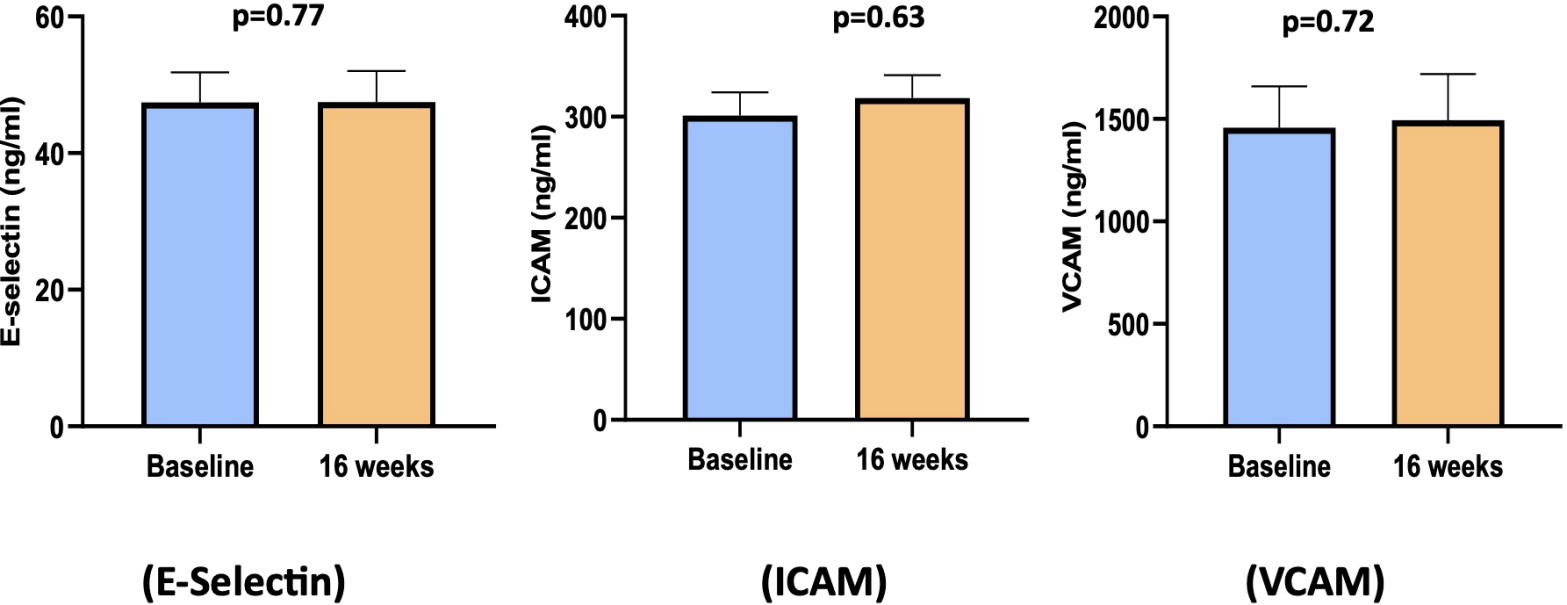
Bar diagram of endothelial cell function biomarkers at baseline and 16 weeks. ICAM, intracellular adhesion molecule; VCAM, vascular cell adhesion molecule.

As shown in **Figure 5**, the circulating levels of endothelial cell function markers (E-Selectin, ICAM, and VCAM) showed no significant change between baseline and 16 weeks.

Abnormal immune response can also lead to increased production and reduced clearance of proinflammatory cytokines, which can cause inflammation and its associated consequences, such as endothelial dysfunction. Thus, we analyzed the association between the change in immune function after cholecalciferol treatment and the change in vascular function (FMD and NMD). No significant correlation was found between the change in immune function (CD3^+^CD4^+^IFNγ^+^, CD3^+^CD4^+^IL4^+^, CD3^+^CD4^+^IL17A^+^, and CD3^+^CD4^+^CD25^+^CD127^low^FOXP3^+^) and the change in vascular function (FMD and NMD) after cholecalciferol supplementation.

## Discussion

This study shows that supplementation with cholecalciferol led to a favorable change in immune function as it favored TH2 cells (CD3^+^CD4^+^IL4^+^cells) and reduced TH1 cells (CD3^+^CD4^+^IFNγ^+^ cells), leading to an overall increased TH2/TH1 ratio, increased expression of vitamin D-responsive genes, and altered the balance of pro- and anti-inflammatory markers in favor of the latter. Cholecalciferol treatment also significantly improved FMD and NMD and showed a trend towards improvement in PWV. However, there was no association between changes in immune and vascular function. Our findings offer direct evidence suggesting that high-dose vitamin D supplementation may have beneficial effects on immune cells, inflammatory markers, and vascular function in CKD.

Vitamin D is linked to a transition from a TH1/TH17 to a TH2 and Treg cell phenotype (20). Vitamin D facilitates a TH2 shift by acting on upstream GATA3 and STAT6 transcription factors (21). We observed that IFN-γ expression on TH1 cells significantly decreased and IL-4, which is a TH2 cell marker, significantly increased after high-dose cholecalciferol treatment. We also observed increased expression of transcription factor STAT6 but not GATA3, which indicates that cholecalciferol directly influences TH cells and can promote the development of a TH2 phenotype. Further, cholecalciferol did not induce changes in the levels of the TH1 transcription factor T-bet, implying that the suppression of IFN-γ is not a result of the inhibition of this crucial transcription factor. The activation of the IL-4 receptor by IL-4, which triggers STAT6 activation, has the potential to guide a naive TH cell toward a TH2 differentiation pathway (22). In most studies, IL-4 production is increased by 1,25(OH)_2_D_3_, although this is not consistent across all studies (23). Our study reported no change in TH17 markers and Treg markers with high-dose cholecalciferol. On the contrary, studies have reported a significant increase in the Treg population with high-dose cholecalciferol in healthy subjects (19). We assume that the absence of a significant impact of cholecalciferol on the percentage of Treg cells might stem from the inadequate responsiveness of Treg cells to activating signals in CKD. It is also uncertain whether the absence of alterations in the proportion of Tregs in peripheral blood cells corresponds to potential changes in Treg cells at the tissue level. A low dose of vitamin D_3_ does affect the natural Treg cell population in healthy adults (24). Aly et al. (25) indicated the low utility of vitamin D in predicting a change in lymphocyte subset as the levels of 25-hydroxyvitamin D_3_ and 1,25(OH)_2_D_3_ showed no association with Tregs or TH17 cells in ESKD patients and renal transplant recipients. Other factors might explain the discrepancies in all these findings of the effect of vitamin D on T cells, such as the state of T cell activation, CYP27B1 activity, and serum concentrations of 25-hydroxyvitamin D_3_ or 1,25(OH)_2_D_3_.

Further, we observed a significant elevation in expression levels of cathelicidin, IL-10, VDR, and CYP27B1 after cholecalciferol supplementation, suggesting nutritional vitamin D could effectively regulate VDR-mediated cellular pathways. Our results are consistent with the findings of Stubbs et al. (26) that cholecalciferol therapy increased monocyte VDR expression and expression of downstream VDR-responsive pathway genes (26). Vitamin D promotes IL-10 production, a characteristic Th2 cytokine, possibly by inducing a shift in CD4+ T-cell phenotype from an inflammatory type (TH17 and TH1) toward a protective (Treg cell and TH2) phenotype (27).

High-dose cholecalciferol supplementation significantly decreased the levels of hsCRP and pro-inflammatory cytokines IFN-γ, TNF-α, IL-6, and IL-23 but did not affect IL-1β, IL-17A, and IL-2. It also upregulated the levels of anti-inflammatory cytokines IL-4, IL-10, and IL-13. Circulating levels of IFN-γ, CRP, IL-6, and TNF-α have been reported to significantly decrease after vitamin D supplementation in patients with CKD and ESRD (26, 28, 29). IL-4, IL-10, and IL-13 levels have been also shown to increase with vitamin D treatment in various clinical conditions (29–31). However, some studies in CKD have reported no significant impact of vitamin D on these cytokines (32–34).

We confirmed the beneficial impact of cholecalciferol supplementation on vascular function as measured by FMD, NMD, and PWV. The findings align with our previously published study that provided robust evidence that correcting vitamin D deficiency enhances FMD and NMD in patients with pre-dialysis CKD (18). These results are also concordant with the study of vitamin D_3_ supplementation in overweight African-American adults (13) and the PENNY Trial that involved paricalcitol supplementation for 12 weeks and reported improvement in FMD patients with stage 3–4 CKD (11).

The levels of E-Selectin, ICAM, and VCAM remained unaffected with cholecalciferol supplementation, supporting the findings of a recent systematic review and meta-analysis of RCTs on metabolic syndrome and related disorders that reported no effect of vitamin D supplementation on E-Selectin, ICAM-1, VCAM-1, and endothelin levels (35). However, there are variable data showing a reduction in E-Selectin, ICAM, and VCAM with high-dose cholecalciferol treatment in non-diabetic patients with CKD (18, 36). These findings suggest that in certain circumstances, an impaired endothelium might exhibit reduced responsiveness, failing to increase production in reaction to vitamin D, unlike in healthy controls or individuals undergoing treatment.

In addition, we noted a significant decline in GFR. In a recent report, vitamin D_3_ supplementation has led to a statistically significant but clinically less meaningful decline in eGFR (37). A few other studies have reported a decrease in eGFR with active vitamin D (38, 39), which may be due to a change in creatinine metabolism rather than an actual decline in GFR. However, the effect of cholecalciferol on creatinine metabolism has not been reported. The increase in urea after cholecalciferol supplementation may be due to the short-term activation of the vitamin D receptor, as it has been shown to reduce kidney function earlier (40). A study on the long-term effect of vitamin D on kidney function is warranted.

In this study, we used two high doses of cholecalciferol that have been shown to be effective for the repletion of vitamin D levels and are safe to use in patients with CKD (18) as we did not observed any adverse events and none of the patients developed hypercalcemia.

Our study has certain strengths: it used a directly observed dose of cholecalciferol to ensure compliance, we used a standardized methodology for FMD measurement, and the exclusion of patients with diabetes led us to assess the impact of cholecalciferol on microcirculation in CKD without the potential confounding effects of diabetes on endothelial function. The limitations include the absence of a placebo group, the single-center design, the relatively short duration of follow-up, and the relatively small sample size. Additionally, we did not record the use of antiepileptics, antivirals, antifungals, and antibiotics in this study, which may have affected the outcome.

In conclusion, cholecalciferol supplementation directly influences immune modulation, as shown by the development of TH2 over TH1 T cells, the decrease in the inflammatory response, and the increase in the vitamin D-responsive gene expression in patients with mild-moderate CKD and vitamin D deficiency. This study also confirms that cholecalciferol supplementation in CKD is effective in improving vascular function. However, we could not demonstrate a link between a change in immune function and a change in vascular function with cholecalciferol supplementation. More studies are needed to understand the relationship between immune function and vascular function and how vitamin D affects the two.

## Data Availability

The original contributions presented in the study are included in the article/**Supplementary Material**, further inquiries can be directed to the corresponding author.
