# Supplementary material for: Effect of cholecalciferol on immune and vascular function in non-diabetic chronic kidney disease
Source: Front Immunol. 2025 Apr 24;16:1555304. doi: 10.3389/fimmu.2025.1555304 (PMC12058780; doi:10.3389/fimmu.2025.1555304)
Supplement: Supplementary file 1 [file Table1.docx]

**Supplementary tables**

| **T Cell phenotype** | **Surface Markers (conjugated dyes)** | **Intracellular Marker (conjugated dyes)** | **Transcription Factor (conjugated dyes)** |
| --- | --- | --- | --- |
| Panel 1: TH1 Cells | CD3(FITC),CD4(BV510), CXCR3(PE) | IFN-γ(APC) | T-bet (BV421) |
| Panel 2: TH2 Cells | CD3(FITC),CD4(BV510), CCR4+(PE), CCR6-(BB700) | IL-4(BV421) | STAT6 (AF647), GATA3 (PE-Cy7) |
| Panel 3: TH17 Cells | CD3(FITC), CD4(BV510), CCR4+(PE), CCR6+(BB700) | IL-17A(BV421) | RORγt (APC) |
| Panel 4: Treg Cells | CD3(FITC), CD4(BV510),  CD25(BV421), CD127^low^(AF647),CD45RA-(PE) | FOXP3(BB700) | FOXP3(BB700) |

**Supplementary Table 1:** Various T cell phenotypes markers and labelled antibodies

AF647: Alexa Fluor 647, APC: [Allophycocyanin (APC)](https://www.aatbio.com/catalog/pe-and-apc#Allophycocyanin), BV421: Brilliant Violet™ 421, BV510: Brilliant Violet™ 510, BB700: Brilliant™ Blue 700, FITC: fluorescein isothiocyanate, FOXP3: forkhead box protein 3, GATA3: GATA binding protein 3, IFN-γ: Interferon-γ, IL-4: Interleukin-4, IL-17A: Interleukin-17A, T-bet: T box transcription factor, TH1: T helper 1 cell population, TH2: T helper 2 cell population, TH17: T helper 17 cell population, Treg: T regulatory cell population, PE: phycoerythrin, , PE-Cy7: phycoerythrin-cyanine7, RORγt: retineic-acid-receptor-related orphan nuclear receptor gamma transcription factor, STAT6: signal transducer and activator of transcription 6

**Supplementary Table 2:** Assay ID and amplicon length of Probes of qRT-PCR experiments

| Gene Name | NCBI reference sequence ID | ABI assay ID | Amplicon length (BP) |
| --- | --- | --- | --- |
| 18s | 18s_consensus.0 | Hs03003631_g1 | 69 |
| Cathelicidin | NM_004345.4 | Hs00189038_m1 | 86 |
| IL-10 | NM_000572.2 | Hs00961622_m1 | 74 |
| VDR | NM_000376.2 | Hs00172113_m1 | 62 |
| CYP27B1 | NM_000785.3 | Hs00168017_m1 | 60 |

BP: basepair, IL-10: Interleukin 10, VDR: vitamin D receptor
